# Markers of inflammation and lung function in liver transplant recipients: results from a nationwide cohort study

**DOI:** 10.3389/fimmu.2026.1804639

**Published:** 2026-04-28

**Authors:** Nicoline Arentoft, Julie Høgh, Hans-Christian Pommergaard, Andreas Dehlbæk Knudsen, Annette Dam Fialla, Paul Suno Krohn, Niels Kristian Aagaard, Jens-Ulrik Stæhr Jensen, Jesper Rømhild Davidsen, Michael Perch, Allan Rasmussen, Susanne Dam Nielsen

**Affiliations:** 1 Department of Infectious Diseases, Copenhagen University Hospital - Rigshospitalet, Copenhagen, Denmark; 2 Department of Clinical Medicine, Faculty of Health and Medical Sciences, University of Copenhagen, Copenhagen, Denmark; 3 Department of Digestive Diseases, Transplantation and General Surgery, Copenhagen University Hospital – Rigshospitalet, Copenhagen, Denmark; 4 Department of Gastroenterology, Odense University Hospital, Odense, Denmark; 5 Department of Hepatology and Gastroenterology, Aarhus University Hospital, Aarhus, Denmark; 6 Department of Respiratory Medicine, Copenhagen University Hospital - Herlev and Gentofte, Gentofte, Denmark; 7 South Danish Center for Interstitial Lung Diseases (SCILS), Department of Respiratory Medicine, Odense University Hospital, Odense, Denmark; 8 Department of Cardiology, Heart and Lung Transplant Unit, Copenhagen University Hospital – Rigshospitalet, Copenhagen, Denmark

**Keywords:** cohort study, comorbidity, inflammation, inflammatory markers, liver transplant recipient, pulmonary disease, spirometry

## Abstract

**Introduction:**

In the general population elevated circulating inflammatory markers have been associated with impaired lung function in cross-sectional and longitudinal studies. No studies have investigated this association in liver transplant recipients, and we aimed to investigate if elevated inflammatory markers were associated with impaired lung function in this population.

**Methods:**

Adult liver transplant recipients from The Danish Comorbidity in Liver Transplant Recipients (DACOLT) study, with available spirometry, high sensitivity (hs)-CRP, interleukin (IL)-1β, IL-2, IL-6, IL-10, interferon (IFN)-γ, and tumor necrosis factor (TNF)-α were included. Outcomes were forced expiratory volume in one second (FEV_1_), forced vital capacity (FVC), airflow limitation, and preserved ratio impaired spirometry (PRISm).

**Results:**

We included 335 liver transplant recipients. The prevalence of airflow limitation and PRISm was 11.6% and 24.5%, respectively. The median FEV_1_ was 2790 mL (IQR 2230–3505 mL) and the median FVC was 3680 mL (IQR 2980–3755 mL). When adjusted for age and sex, hs-CRP >3 mg/L was associated with increased odds of PRISm (aOR 2.08, 95% CI: 1.1; 3.9, p=0.02), lower FEV_1_ (-209 mL 95% CI -340; -77 mL, p<0.01), and lower FVC (-290 mL 95% CI -448 mL; -132 mL, p<0.01). For FEV_1_ and FVC, the associations were consistent when additionally adjusted for ethnicity, BMI, and smoking status. None of the remaining inflammatory markers were significantly associated with any of the outcomes across statistical models.

**Conclusion:**

Elevated hs-CRP was associated with impaired lung function indicating that systemic inflammation may be part of the pathophysiology of impaired lung function in liver transplant recipients.

## Introduction

Liver transplantation is the only curable treatment for end-stage liver disease. Due to the extensive immunosuppressive medication, liver transplant recipients are at increased risk of pulmonary infections (1–4). However, little is known about the burden of chronic pulmonary diseases in this vulnerable population. We previously found that living with a transplanted liver was associated with increased eosinophilic airway inflammation measured as fraction of exhaled nitric oxide (5). Furthermore, we found that liver transplant recipients had double the risk of airflow limitation, and lower forced expiratory volume in 1 second (FEV_1_) and forced vital capacity (FVC) than matched controls from the general population (6). Similar findings regarding airflow limitation have been observed in kidney transplant recipients suggesting that lung function impairment may be a significant comorbidity within solid organ transplantation (7). This warrants further investigation of the underlying mechanisms leading to lung function impairment in liver transplant recipients.

Several circulating markers of systemic inflammation, including C-reactive protein (CRP) or high-sensitivity (hs)-CRP, tumor necrosis factor α (TNF- α), and interleukin (IL)-6, have been linked to reduced lung function and chronic obstructive pulmonary disease (COPD) in the general population (8–12). Thus, systemic inflammation, and not just pulmonary inflammation, may play a mechanistic role in development of lung function impairment. One recent study found that liver transplant recipients had higher levels of systemic inflammation than controls measured with a composite inflammatory marker and another study found that liver transplant recipients had higher levels of hs-CRP than controls (13, 14). The presence of increased systemic inflammation in this population makes liver transplant recipients a particularly relevant cohort in which to examine the relationship between systemic inflammation and lung function impairment.

Thus far, studies of circulating inflammatory markers in liver transplant recipients have focused on identifying immunomodulatory cells that are associated with graft survival and on finding inflammatory markers that may predict graft rejections (15, 16). Whether certain circulating inflammatory markers can be linked to an increased risk of impaired lung function in liver transplant recipients is unknown, and to our knowledge no studies on circulating inflammatory markers and lung function in liver transplant recipients exist.

The aim of this study was to investigate if circulating inflammatory markers were associated with airflow limitation, preserved ratio impaired spirometry (PRISm), FEV_1_, and FVC in liver transplant recipients. We hypothesize that elevated circulating inflammatory markers are associated with increased risk of airflow limitation and PRISm and with lower FEV_1_ and FVC.

## Methods and materials

### Study design and population

This study utilized cross-sectional baseline data from The Danish Comorbidity in Liver Transplant Recipients (DACOLT) study. The DACOLT study is a nationwide prospective cohort study initiated in March 2021. By June 2025, the DACOLT study had included >75% of eligible liver transplant recipients in Denmark. The overall purpose of the DACOLT study is to investigate the prevalence, incidence, and pathogenesis of comorbidities in liver transplant recipients (17). All adult liver transplant recipients (>20 years) who could give informed consent and were followed at one of the four Danish outpatient clinics for liver transplant recipients: Copenhagen University Hospital – Rigshospitalet, Aarhus University Hospital, Odense University Hospital, and Aalborg University Hospital, were invited to participate in the DACOLT study. This study includes participants, who were included before or on 31^st^ of May 2023 and had performed spirometry and had available measurements of plasma hs-CRP, IL-1β, IL-2, IL-6, IL-10, IFN-γ, and TNF-α.

All participants answered a detailed questionnaire on lifestyle, medical history, and medication, and underwent a physical examination, including spirometry, which was performed by trained healthcare professionals.

This study was reported in adherence with Strengthening the Reporting of Observational Studies in Epidemiology (STROBE) guidelines.

### Ethics statement

The DACOLT study (Clinical Trials identifier: NCT04777032) was approved by the Committee on Health Research Ethics of the Capital Region of Denmark (approval number H-20052199). Written informed consent was obtained from all participants. The study was conducted according to the Declaration of Helsinki.

### Spirometry and physical examination

An EasyOne spirometer (ndd Medical, Zürich, Switzerland) was used for spirometries. Trained healthcare professionals instructed participants in accordance with American Thoracic Society/European Respiratory Society guidelines except participants were in a standing position and did not use a nose clip (18). Participants were instructed to 1) maximum inspiration, 2) blow forcefully in the beginning of the exhalation, and 3) continue exhalation until the end of the spirometry.

Only pre-bronchodilator spirometries were performed. Predicted values of FEV_1_ and FVC were calculated using the multiethnic reference equations stated by the Global Lung Function Initiative (19). Preserved ratio impaired spirometry (PRISm) was defined as FEV_1_/FVC ≥ 0.7 and FEV_1_ <80% of expected and airflow limitation was defined by the fixed criterion as FEV_1_/FVC <0.7 and predicted FEV_1_ <80% as recommended by The Global Initiative for Chronic Obstructive Lung Disease (GOLD) 2025 report (20).

As part of the physical examination, the participants’ height and weight were measured. Body mass index (BMI) was calculated as weight in kilograms/height in meters^2^ and categorized according to the World Health Organization recommendations as: underweight: <18.5, normal weight: 18.5-24.9, overweight: 25.0-30.0, and obese: >30.0 (21).

### Inflammatory markers

At inclusion in the DACOLT study, participants had blood drawn for both routine biochemistry, including hs-CRP, and plasma was separated and stored in a biobank at -80°C.

Hs-CRP was analyzed using turbidimetric assay at Department of Clinical Biochemistry at Copenhagen University Hospital – Rigshospitalet.

Plasma concentrations of IL-1β, IL-2, IL-6, IL-10, IFN-γ, and TNF-α were measured using the electrochemiluminescence-based sandwich immunoassay Meso Scale Discovery (MSD) V-PLEX Proinflammatory Panel 1 Human Kit (Meso Scale Diagnostics LLC, Maryland, USA) at Department of Clinical Immunology at Copenhagen University Hospital – Rigshospitalet, according to the manufacturer’s instructions (22). All panels were analyzed on the Meso QuickPlex SQ 120 platform (Meso Scale Diagnostics LLC, Maryland, USA). Duplicates of internal control were analyzed and a standard curve was yielded. Using only dilutent, a negative control was performed” (22).

Inflammatory markers were dichotomized using a pre-specified cut-off. Hs-CRP was dichotomized using >3 mg/L as cut-off reflecting systemic inflammation as previously recommended (23). All other inflammatory markers were dichotomized using the third quartile as cut-off as previously done (24, 25).

### Self-reported variables

Information on asthma, chronic obstructive pulmonary disease (COPD), educational status, and smoking was collected from structured questionnaires. Asthma was defined as a “yes” to the question: “Do you have asthma?” (26). COPD was defined as a “yes” to the question: “has a doctor or a nurse ever told you, that you have COPD?”. Educational status was defined as the longest completed education grouped as: no education, short education (<3 years), vocational education, middle length education (>3 years, e.g., teacher, nurse, or similar), or university degree. Cumulative smoking was measured in pack-years, given as the number of years an individual had smoked 20 cigarettes per day.

Ethnicity was based on the origin of the participants grandparents and grouped as “Caucasian” and “other”.

### Variables from medical records

Liver transplant related variables were collected from medical records. Time since transplantation was defined as time from first liver transplantation to time of inclusion in the DACOLT study. Acute rejection was defined as having one or more biopsy-verified acute rejections treated with 1000 mg methylprednisolone for 3–5 days. Cirrhosis at time of transplantation was affirmative a pathology description confirmed cirrhosis in a biopsy from the liver explant. Data on immunosuppressive medication was collected from the national Danish Shared Medication Record (FMK), which contains information about all medications prescribed in Denmark (27).

### Statistics

Distribution of data was assessed using histograms. Normally distributed variables were summarized with means and standard deviations and non-normally distributed variables were summarized with medians and interquartile ranges. Categorical variables were described as frequencies and percentages.

To investigate if there was an association between inflammatory markers and the binary outcomes airflow limitation and PRISm, multivariable logistic regression was used. To investigate if there was an association between inflammatory markers and the continuous outcomes FEV_1_ and FVC, multivariable linear regression was used. Model assumptions were tested using QQ-plots, histograms, and residual plots. Possible confounders included in the models were decided upon *a priori* based upon existing literature. Analyses were done with a minimally adjusted model 1 adjusted for age and sex and a fully adjusted model 2 adjusted for age, sex, ethnicity, BMI, and smoking status. The inflammatory markers were tested one at a time.

For hs-CRP, the following sensitivity analyses were performed: i) excluding those with hs-CRP >10 mg/L to account for possible concurrent acute infection which could influence the spirometry performance and ii)only including participants in current treatment with prednisolone were performed to account for the possible influence of prednisolone.

In exploratory analyses, the association between tacrolimus trough levels within 3 months before or after study inclusion and elevated circulation inflammatory markers was investigated using multivariable logistic regression adjusted for age and sex. Tacrolimus trough levels was log2 transformed. In exploratory analyses we investigated if there was an interaction between smoking status and elevated inflammatory markers on significant associations with FEV_1_ and FVC.

P-values ≤0.05 were considered statistically significant. All analyses were conducted using R (version 4.3.0).

## Results

### Study population

We included 335 liver transplant recipients from the DACOLT study with a median age of 56 years (**Table 1**). Less than half of the participants were female (43%) and there was a predominance of Caucasian decent (93%). Almost 13% were current smokers at time of inclusion.

**Table 1:** Characteristics, lung related variables, and liver transplant variables.

Liver transplant recipients (n=335)	
Characteristics.	
Sex (female), n (%)	144 (43.0%)
Age, years, median (IQR)	55.7 (46.8-64.5)
Ethnicity, n, (%)	
- Caucasian - Other	311 (92.8) 24 (3.7)
Height, cm, median (IQR)	171.7 (165.9-179.1)
Weight, kg, median (IQR)	79.8 (69.6-90.6)
Body mass index, median (IQR)	26.9 (24.0-30.1)
Smoking status, n (%)	
- Current - Previous - Never	43 (12.8) 118 (35.2) 167 (49.9)
Smoking history, pack-years, median (IQR)	10.0 (4.0-20.0)
Hs-CRP >10 mg/L, n, (%)	43 (12.8)
Lung related variables	
Self-reported asthma, n (%)	24 (7.2)
Self-reported COPD, n (%)	7 (2.1)
Use of inhalation medicine for asthma or bronchitis at baseline, n (%)	23 (6.9)
FEV_1_, mL, median, (IQR)	2790 (2230-3505)
FVC, mL, median, (IQR)	3680 (2980-3755)
FEV_1_/FVC<0.7, n (%)	56 (16.7)
PRISm, n (%)	82 (24.5)
Airflow limitation, n (%)	39 (11.6)
*Liver transplant variables*	
Time since transplantation, years, median (IQR)	7.1 (2.7-13.4)
- Reason for transplantation*, n (%)	
- Autoimmune liver disease	146 (43.6)
o Autoimmune hepatitis	40
o Primary sclerosing cholangitis	99
o Primary biliary cholangitis	25
- Alcohol-associated or cryptogenic cirrhosis	68 (20.3)
- Hepatocellular carcinoma	23 (6.9)
- Fulminant hepatic failure	24 (7.2)
- Metabolic disease	17 (5.1)
- Hepatitis C	13 (3.9)
- Biliary atresia	11 (3.3)
- Metabolic dysfunction-associated steatohepatitis	11 (3.3)
- Hepatitis B	9 (2.7)
- Other	29 (8.7)
Cirrhosis, any, at time of transplantation, n (%)	223 (66.6)
Acute rejection, ever, n (%)	41 (12.2)
Immunosuppressive medication, n (%)	
- Prednisolone	161 (48.1)
- Tacrolimus	276 (83.3)
- Ciclosporin	35 (10.4)
- Everolimus	21 (6.3)
- Mycophenolate mofetile	245 (73.1)
- Azathioprine	31 (9.3)

The most common reason for transplantation was autoimmune liver disease (44%) and tacrolimus was the predominant immunosuppressive medication (81%) followed by mycophenolate mofetile (73.1%) and prednisolone (48.1%).

### Elevated circulating inflammatory markers and airflow limitation and PRISm

**Figure 1:**
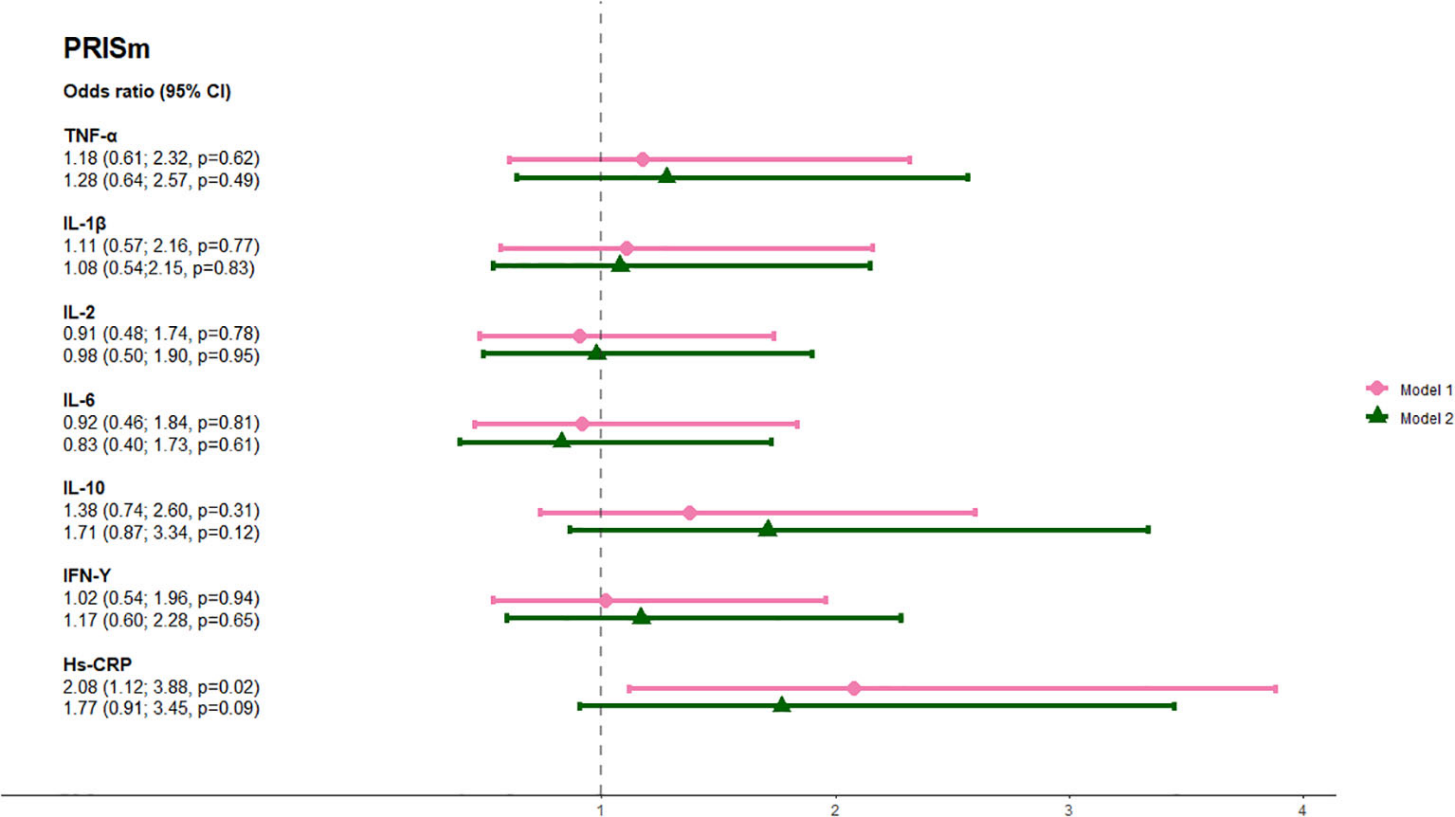
Association between circulating inflammatory markers and preserved ratio impaired spirometry (PRISm). Analyses were done with a minimally adjusted model 1 adjusted for age and sex and a fully adjusted model 2 adjusted for age, sex, ethnicity, BMI, and smoking status. Pink lines show results adjusted for Model 1 and green lines show results adjusted for Model 2. aORs are shown on the far left. Abbreviations: TNF, tumor necrosis factor; IL, interleukin; IFN, interferon; hs-crp, high-sensitivity CRP.

**Figure 2:**
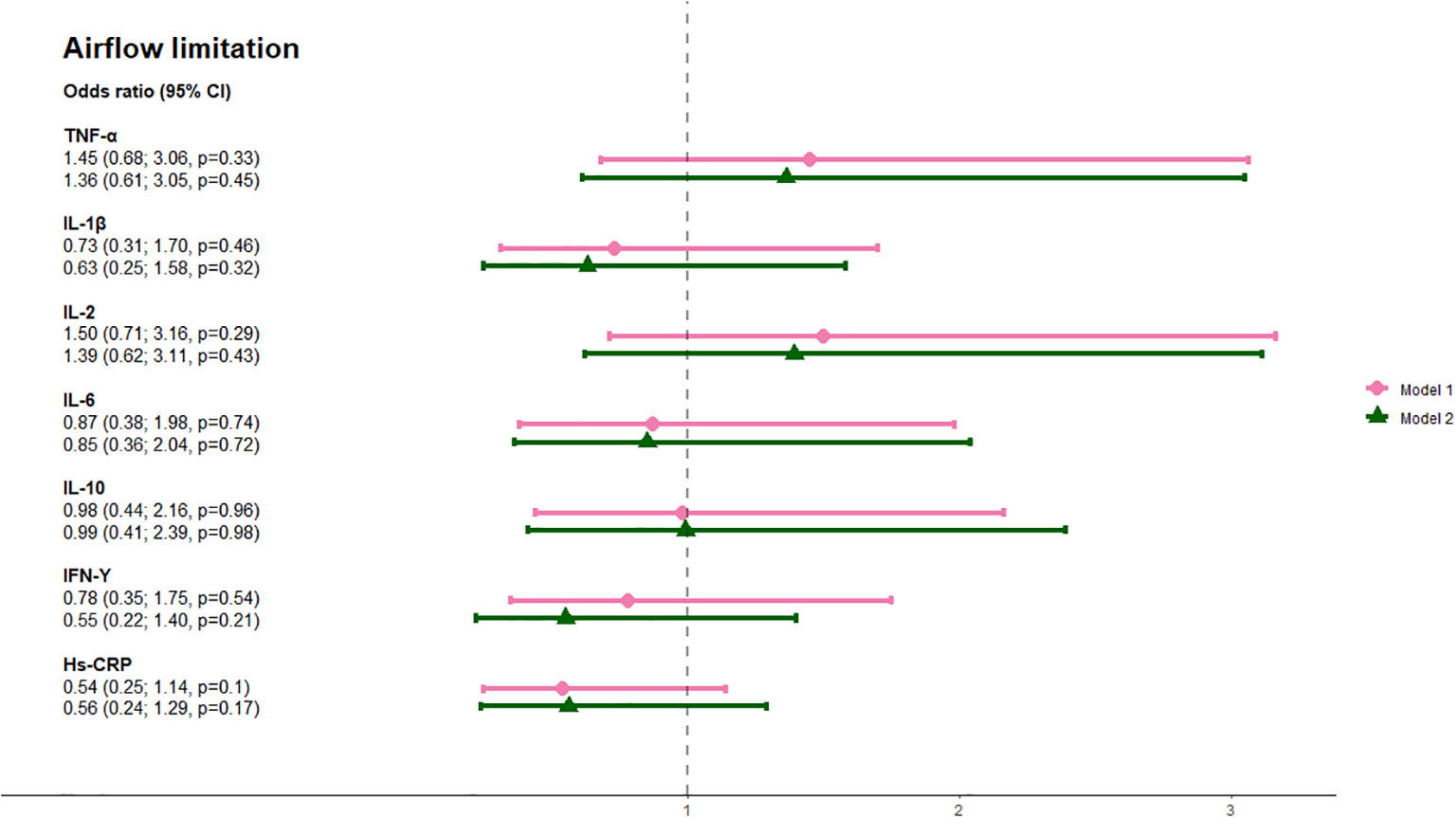
Association between circulating inflammatory markers and airflow limitation. Analyses were done with a minimally adjusted model 1 adjusted for age and sex and a fully adjusted model 2 adjusted for age, sex, ethnicity, BMI, and smoking status. Pink lines show results adjusted for Model 1 and green lines show results adjusted for Model 2. aORs are shown on the far left. Abbreviations: TNF, tumor necrosis factor; IL, interleukin; IFN, interferon; hs-crp, high-sensitivity CRP.

The prevalence of airflow limitation and PRISm was 11.6% and 24.5%, respectively (**Table 1**). Median values and interquartile ranges (IQR) of the inflammatory markers are displayed in **Table 2**. Elevated hs-CRP (>3.0 mg/L) was associated with a twofold increase in the odds of PRISm when adjusted for model 1 (aOR 2.08, 95% CI: 1.1; 3.9, p=0.02). This association was attenuated, and no longer statistically significant, when further adjusted for model 2 (aOR 1.77, 95% CI: 0.9; 3.5, p=0.09). None of the other inflammatory markers were associated with PRISm (**Figure 1**). None of the inflammatory markers were significantly associated with higher odds of airflow limitation in either the minimally adjusted model 1, adjusted for age and sex, or the fully adjusted model 2, additionally adjusted for ethnicity, BMI, and smoking status (**Figure 2**).

**Table 2:** Inflammatory markers.

Inflammatory marker	Median (IQR)
TNF-α, pg/mL,	1.48 (1.16-1.97)
IL-1β, pg/mL,	0.09 (0.04-0.20)
IL-2, pg/mL,	0.39 (0.20-0.60)
IL-6, pg/mL,	1.39 (0.82-2.42)
IL-10, pg/mL,	0.36 (0.21-0.68)
IFN-γ, pg/mL,	7.92 (5.07-15.20)
Hs-crp, mg/L	2.46 (1.17-5.37)

### Elevated circulating inflammatory markers and lung function

**Figure 3:**
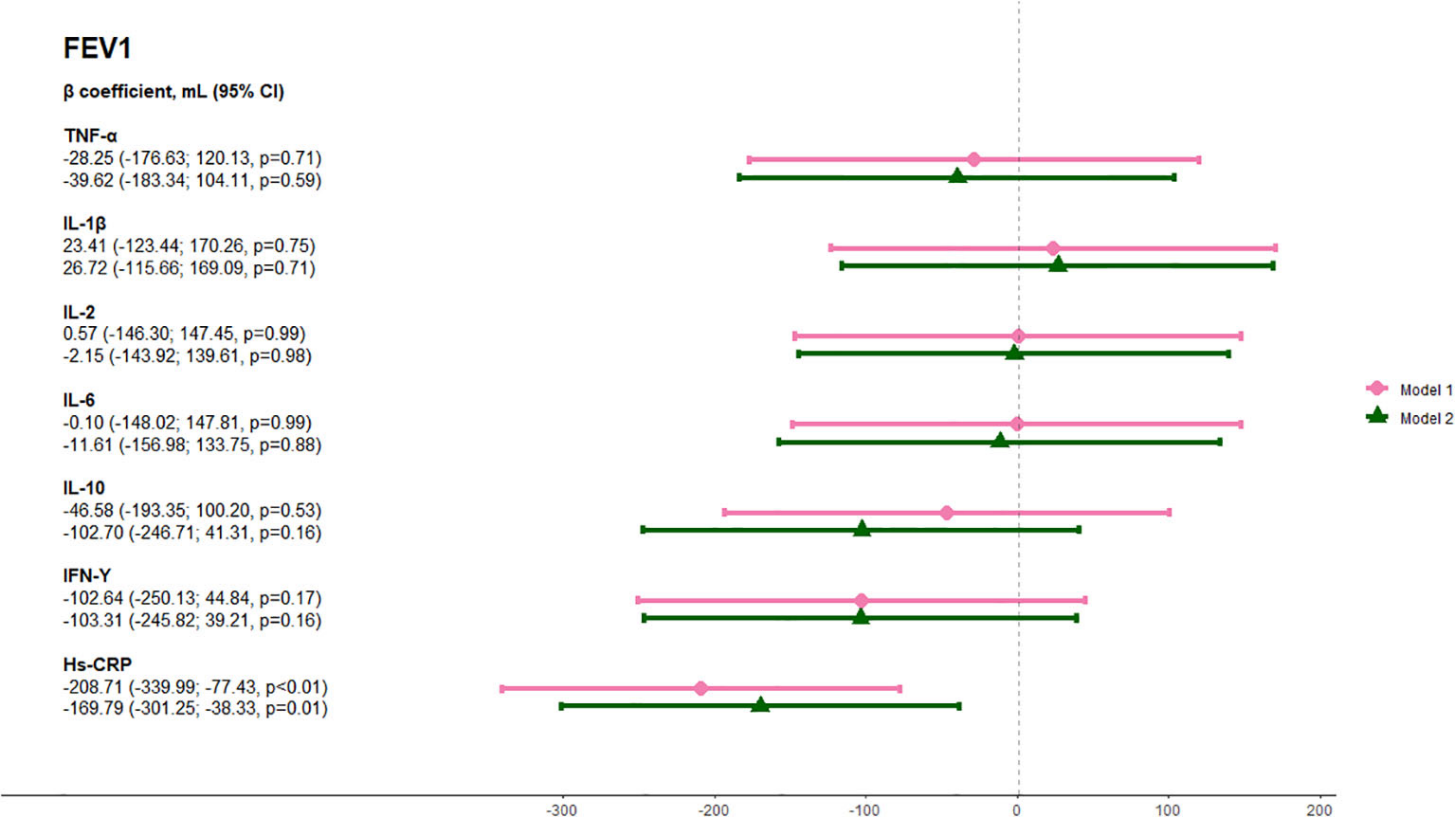
Association between circulating inflammatory markers and forced expiratory volume in one second (FEV_1_). Analyses were done with a minimally adjusted model 1 adjusted for age and sex and a fully adjusted model 2 adjusted for age, sex, ethnicity, BMI, and smoking status. Pink lines show results adjusted for Model 1 and green lines show results adjusted for Model 2. B coefficients are shown on the far left. Abbreviations: TNF, tumor necrosis factor; IL, interleukin; IFN, interferon; hs-crp, high-sensitivity CRP.

**Figure 4:**
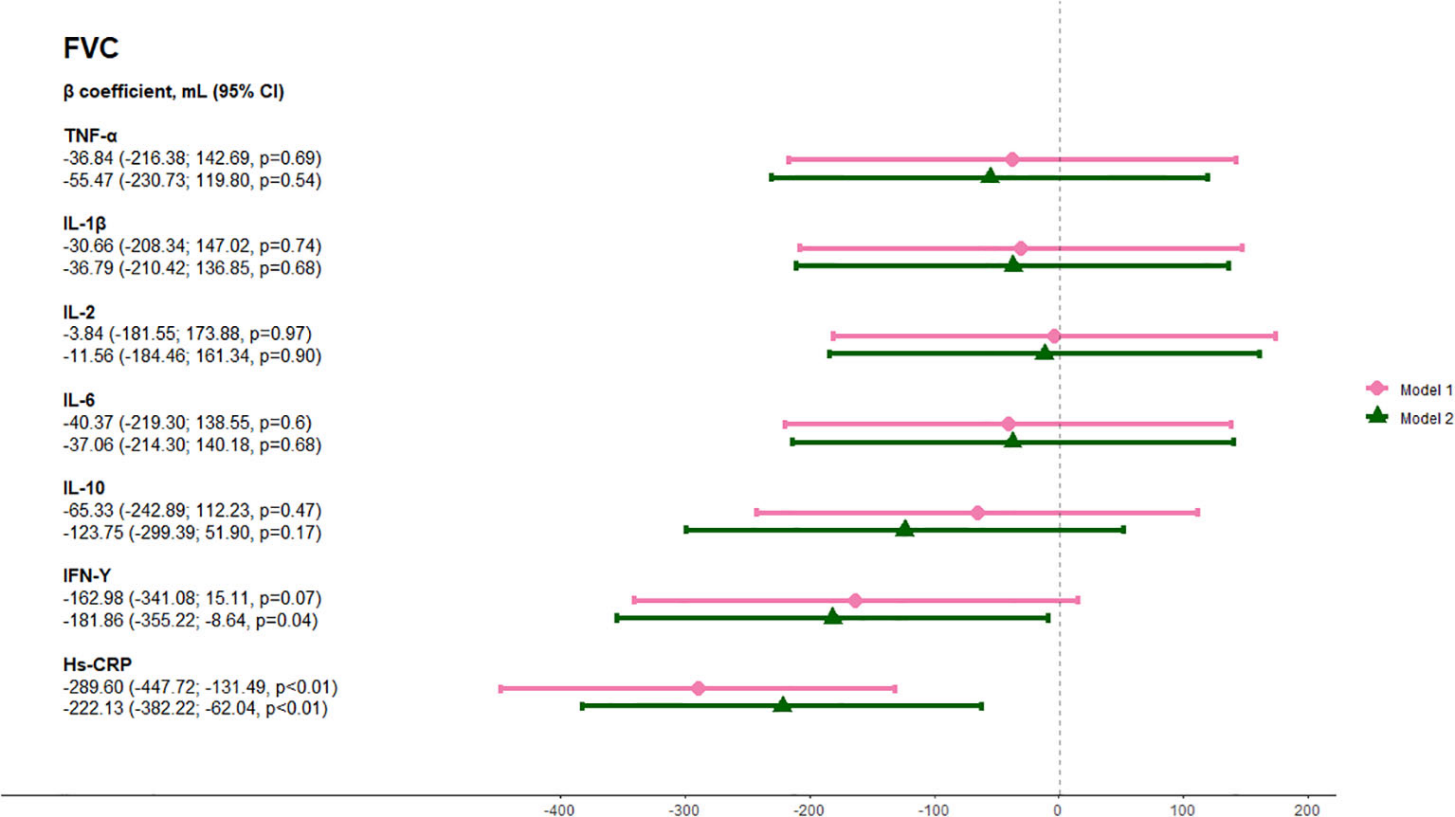
Association between circulating inflammatory markers and forced vital capacity (FVC). Analyses were done with a minimally adjusted model 1 adjusted for age and sex and a fully adjusted model 2 adjusted for age, sex, ethnicity, BMI, and smoking status. Pink lines show results adjusted for Model 1 and green lines show results adjusted for Model 2. B coefficients are shown on the far left. Abbreviations: TNF, tumor necrosis factor; IL, interleukin; IFN, interferon; hs-crp, high-sensitivity CRP.

The median FEV_1_ was 2,790 mL (IQR 2,230-3,505 mL) and the median FVC was 3,680 mL (IQR 2,980-3,755 mL). Elevated hs-CRP was associated with 208.7 mL lower FEV_1_ (95% CI -340.0; -77.4 mL, p<0.01) when adjusted for model 1 and 169.8 mL lower FEV_1_ (95% CI -301.3 mL; -38.3 mL, p<0.01) when further adjusting for model 2 (**Figure 3**). Likewise, elevated hs-CRP was associated with 289.6 mL lower FVC (95% CI -447.7 mL; -131.5 mL, p<0.01) when adjusting for model 1 and 222.1 mL lower FVC (95% CI -382.2 mL; -62.0 mL, p<0.01) when adjusting for model 2. Furthermore, IFN-γ was associated with 181.9 mL lower FVC (95% CI -355.1 mL; -8.6 mL, p=0.04) in the fully adjusted model 2. None of the other inflammatory markers were significantly associated with lung function (**Figure 4**). There was an interaction between smoking status and elevated hs-CRP on the association with both FEV_1_ (p-interaction=0.0006) and FVC (p-interaction=0.006).

### Sensitivity analyses

To account for potential acute infections, sensitivity analyses were performed excluding participants with hs-CRP >10 mg/L. In this subgroup, results were consistent with no significant association between elevated hs-CRP and airflow limitation while elevated hs-CRP was associated with PRISm (aOR 2.16, 95% CI 1.0; 4.66, p <0.05) in the fully adjusted model. Furthermore, the associations with lower FEV1 (-185.1 mL (95% CI -326.9 mL; -43.3 mL, p=0.01) and lower FVC (-234.0 mL 95% CI -408.5; -59.6 mL, p <0.01) remained consistent and significant (**Table 3**). Furthermore, analyses only including participants in current treatment with prednisolone were performed. In this subgroup, the direction of the associations remained consistent as did the magnitude of the odds ratios and β coefficients, however only the association between hs-CRP and FVC adjusted for Model 1 remained statistically significant (-242.36, 95% CI -477.34; -7.38, p=0.04) (**Table 3**).

**Table 3:** Sensitivity analyses regarding associations between hs-CRP and lung function measures.

Association	Model 1		Model 2	
Excluding participants with hs-CRP >10 mg/L				
FEV1	β coefficient, mL, 95%CI	*P*	β coefficient, mL, 95%CI	*P*
Hs-crp	-211.34 (-352.57; -70.20)	0.003	-185.11 (-326.91; -43.32)	0.01
FVC	β coefficient, mL, 95%CI	*P*	β coefficient, mL, 95%CI	*P*
Hs-crp	-287.82 (-458.86; -116.77)	0.001	-234.04 (-408,50; -59.59)	<0.01
Airflow limitation	aOR, 95%CI	*P*	aOR, 95%CI	*P*
Hs-CRP	0.42 (0.17; 1.04)	0.06	0.42 (0.15; 1.17)	0.1
PRISm	aOR, 95%CI	*P*	aOR, 95%CI	*P*
Hs-CRP	2.31 (1.13; 4.72)	0.22	2.16 (1.00; 4.66)	<0.05
Including only participants in current treatment with prednisolone				
FEV1	β coefficient, mL, 95%CI	*P*	β coefficient, mL, 95%CI	*P*
Hs-crp	-172.18 (-360.83; 16.47)	0.08	-180.86 (-369.78; 8.06)	0.06
FVC	β coefficient, mL, 95%CI	*P*	β coefficient, mL, 95%CI	*P*
Hs-crp	-242.36 (-477.34; -7.38)	0.04	-226.81 (-460.87; 7.25)	0.06
Airflow limitation	aOR, 95%CI	*P*	aOR, 95%CI	*P*
Hs-CRP	0.32 (0.08; 1.28)	0.11	0.46 (0.11; 1.94)	0.29
PRISm	aOR, 95%CI	*P*	aOR, 95%CI	*P*
Hs-CRP	2.16 (0.87; 5.38)	0.10	2.05 (0.77; 5.41)	0.14
Only including never smokers				
FEV1	β coefficient, mL, 95%CI	*P*	β coefficient, mL, 95%CI	*P*
Hs-crp	-190.01 (-326.39; -17.62)	0.03	-150.94 (-324.19; 22.31)	0.09
FVC	β coefficient, mL, 95%CI	*P*	β coefficient, mL, 95%CI	*P*
Hs-crp	-259.21 (-476.83; -41.58)	0.02	-192.13 (-407.16; 22.89)	0.08
Airflow limitation	aOR, 95%CI	*P*	aOR, 95%CI	*P*
Hs-CRP	0.40 (0.12;1.34)	0.14	0.41 (0.12; 1.44)	0.16
PRISm	aOR, 95%CI	*P*	aOR, 95%CI	*P*
Hs-CRP	2.75 (1.04; 7.26)	0.04	2.45 (0.89; 6.74)	0.08
Only including ever smokers				
FEV1	β coefficient, mL, 95%CI	*P*	β coefficient, mL, 95%CI	*P*
Hs-crp	-212.15 (-408.47; -15.83)	0.04	-177. 20 (-379.03; 24.62)	0.09
FVC	β coefficient, mL, 95%CI	*P*	β coefficient, mL, 95%CI	*P*
Hs-crp	-305.69 (539.38; -72.00)	0.01	-242.16 (-481.41; -2.90)	<0.05
Airflow limitation	aOR, 95%CI	*P*	aOR, 95%CI	*P*
Hs-CRP	0.61 (0.22; 1.72)	0.34	0.77 (0.26; 2.34)	0.64
PRISm	aOR, 95%CI	*P*	aOR, 95%CI	*P*
Hs-CRP	1.61 (0.69; 3.74)	0.27	1.34 (0.55; 3.30)	0.52

Model 1: adjusted for age and sex. Model 2: Adjusted for age, sex, smoking, BMI, and ethnicity.

### Tacrolimus trough levels and inflammatory markers

In analyses of the association between tacrolimus trough levels and elevated circulating inflammatory markers, every doubling of tacrolimus trough level was associated with an aOR of 1.61 (95% CI 1.00; 2.59, p<0.05) for elevated IL-1β and an aOR of 3.32 (95% CI 1.95; 5.65, p<0.001) for elevated IL-10. Higher tacrolimus trough levels were not significantly associated with any other circulating inflammatory markers (**Table 4**).

**Table 4:** association between log2 transformed tacrolimus trough levels and elevated circulating inflammatory markers.

Inflammatory marker	aOR (95% CI)	*P*
IL-1β	1.61 (1.00; 2.59)	<0.05
IL-2	0.71 (0.45; 1.12)	0.14
IL-6	1.19 (0.74; 1.93)	0.47
IL-10	3.32 (1.95; 5.65)	<0.001
TNF-α	0.78 (0.49; 1.26)	0.32
IFN-γ	1.08 (0.69; 1.71)	0.74
Hs-CRP	1.10 (0.73; 1.66)	0.65

OR adjusted for age and sex.

## Discussion

In this large nationwide cohort study with cross-sectional baseline data, we investigated possible associations between circulating inflammatory markers IL-1β, IL-2, IL-6, IL-10, IFN-γ, TNF-α, and hs-CRP and different lung function measures in liver transplant recipients. The study demonstrated that liver transplant recipients with elevated hs-CRP had a higher risk of PRISm and profoundly lower FEV_1_ and FVC after a median time since transplantation of 7 years.

Our group recently discovered that liver transplant recipients have higher odds of impaired lung function when compared to the general population (6). As chronic pulmonary disease in liver transplantation is an emerging research area, the mechanisms leading to impaired lung function in this population is unknown. Impaired lung function in the general population is a result of damage to the airways or lung tissue often due to smoking, but environmental and genetic factors, infections, and, potentially, systemic inflammation also play a role in the underlying pathophysiology (20, 28). Thus, in the general population both cross-sectional and longitudinal studies have demonstrated an inverse association between presence of circulating inflammatory markers and lung function (8–10, 29). Even though the association between systemic inflammation and lung function impairment have been established in the general population, it is highly relevant to investigate if the association exists in immunosuppressed populations with previously reported higher inflammatory burden (13, 14). Thus, this study extends the existing knowledge about circulating inflammatory markers and lung function to also include liver transplant recipients receiving concurrent systemic immunosuppressive medication.

We found that hs-CRP >3 mg/L was significantly associated with PRISm, lower FEV_1_, and lower FVC but not with airflow limitation. The differences in FEV_1_ and FVC between the groups with elevated hs-CRP and normal hs-CRP was 170 mL and 220 mL, respectively, even after adjusting for possible confounders. Assuming a physiological age-related FEV_1_ decline of 30 mL/year (30), this is equivalent to more than five years of additional aging. This suggests systemic inflammation as reflected by hs-CRP may have a clinically relevant impact on lung function in liver transplant recipients. Consistent with our findings, a large study in the general population (n=7753) found elevated CRP to be associated with lower FEV_1_ and FVC but not FEV_1_/FVC (8). Interestingly, we found an interaction between smoking status and elevated hs-CRP on the association between elevated hs-CRP and lower FEV_1_ and FVC. This is in line with existing literature illustrating that smoking may cause both increased inflammation and impaired lung function (20). CRP is produced in the liver and may have a protective effect on acute liver injury (31, 32). Hs-CRP, along with the less sensitive CRP, is a widely used acute phase reactant and biomarker of inflammation and tissue injury and has long been employed by clinicians when evaluating patients with infections (33). The clinical application of hs-CRP has been expanded to include the assessment of low-grade systemic inflammation and cardiovascular risk stratification (34, 35). Additionally, CRP is closely linked to the prognosis of a series of diseases including cardiovascular disease and cancers (31). Recently, hs-CRP was found to be elevated in liver transplant recipients compared to controls and associated with all-cause mortality (14). In the context of chronic pulmonary disease, the association between impaired lung function and inflammation is likely bidirectional (10, 36, 37). Systemic inflammation may drive tissue damage and lung function decline, but declining lung function may also predict higher CRP levels. Although the temporal direction of this relationship remains difficult to unfold and the interplay between systemic inflammation and impaired lung function is complicated, the evidence suggests that the two are closely linked. Although the efficacy of monitoring hs-CRP to predict pulmonary complications have not been established, elevated hs-CRP may correlate with increased mortality in patients with COPD (38). As hs-CRP is a widely used and inexpensive biomarker performed as part of routine care at outpatient clinics when evaluating liver transplant recipients, further studies of its clinical efficacy could be of great value to the patients.

We found that elevated IFN- γ was associated with a 180 mL lower FVC in the fully adjusted model, but not with lower FEV_1_ or with PRISm and airflow limitation. IFN-γ is a diverse cytokine that may both induce, mainly by macrophage activation, and regulate the inflammatory response. This contradiction is evident in the literature on IFN-γ and lung function. While elevated IFN-γ was more prevalent in patients with asthma than in controls in one study (39) and IFN- γ producing Th17/Th1 cells have been found in patients with COPD (40), IFN-γ may also have modulatory effects which help protect against airflow obstruction (41) and to limit inflammatory-driven tissue damage (42). Thus, the observed association between elevated IFN-γ and reduced FVC in liver transplant recipients may reflect inflammation-related lung function impairment or, alternatively, a compensatory IFN-γ response to limit cytokine-induced tissue damage.

Surprisingly, we did not find any associations between circulating inflammatory markers and lung function besides hs-CRP and IFN-γ. In previous studies of non-transplanted individuals, elevated IL-6 and TNF-α have been linked to poorer lung function (11, 12, 25). Since CRP is a downstream product of IL-6 the two could be expected to have similar associations with lung function and therefore it is especially surprising that we did not find an association for IL-6. This discrepancy could be due to several factors: CRP is more stable than IL-6 and consequently more reliable in studies including only one measurement (43, 44), CRP synthesization is influenced by other cytokines in addition to IL-6 and thus could be seen as a broader marker of inflammation (44), and IL-6 pathways, together with other markers of inflammation measured in this study including IL-2, TNF-α, and IFN-γ, may be affected by tacrolimus, a calcineurin-inhibitor prescribed to more than 80% of the participants in our study, which could alter the composition and concentrations of inflammatory markers in liver transplant recipients (45, 46).

Airway inflammation is present in patients with COPD or asthma and is a part of the pathophysiology behind these conditions (20, 47, 48). In line with this, we previously found that living with a transplanted liver was associated with higher levels of fraction of exhaled nitric oxide than in the general population, thus indicating that airway inflammation is more often present in liver transplant recipients (5). The relationship between local inflammation in the airways and systemic inflammation is complex and the underlying mechanism behind systemic inflammation and lung function impairment may be multifactorial (49, 50). Because the entire cardiac output, carrying all circulating inflammatory markers, passes through the pulmonary circulation, the lung endothelium is continuously exposed to systemic inflammatory signals (51). In response, endothelial permeability may increase, facilitating translocation of pathogens and immune cells between the airways and the bloodstream, where they can trigger additional immune activation and thereby amplify the systemic inflammation (52, 53). Alveolar macrophages can induce both local pulmonary inflammation and systemic inflammation in response to inhaled particles, cytokines produced by alveolar macrophages have shown to be present in circulation and human pulmonary epithelial cells can produce CRP (52, 54–56). Airway inflammation may lead to endothelial damage, breakdown of extracellular matrix leading to emphysema, tissue damage and tissue remodeling, and pulmonary fibrosis resulting in reduced gas exchange all of which could result in impaired lung function and pulmonary morbidity (57–59). Thus, the lungs may be not only a target of inflammation but also a potential amplifier of the inflammatory cascade. Liver transplant recipients, who are particularly vulnerable to airway infections due to chronic immunosuppressive medication and who may exhibit elevated levels of systemic inflammation compared with non-transplanted individuals, could therefore be especially susceptible to this bidirectional amplification loop between systemic inflammation and pulmonary responses (1, 4, 13, 14).

Liver transplant recipients are treated with immunosuppressive medication to minimize the risk of graft rejection. Our analyses of tacrolimus trough levels showed an increased risk of elevated IL-1β and IL-10, but no other significant associations were identified. As calcineurin inhibitors are known to suppress IL-2, we were surprised not to find a negative association, but this may be due to the short half-life of IL-2 (60). These effects of immunosuppressive treatment on circulating inflammatory markers are, to our knowledge, not previously reported, and additional studies could provide valuable insights.

This study had some important limitations. First, the cross-sectional nature of the study prevents any temporal or causal interpretations. Thus, based on the current results, we cannot conclude whether systemic inflammation may lead to impaired lung function, or if impaired lung function can result in systemic inflammation in liver transplant recipients. Nonetheless, findings comparable to ours from cross-sectional studies in the general population have been confirmed by longitudinal research (10, 29). Second, the most common reason for transplantation in our cohort was autoimmune liver disease, which reflects the liver transplant population in Denmark and Scandinavia but differs from the global liver transplant population. Therefore, the results should be validated in a different cohort.

Strengths of the study include the large number of participants which allowed adjustment for possible confounders and the diverse panel of inflammatory markers. The study population is well-described and study inclusion was performed by trained healthcare professionals with spirometry experience following a strict protocol which secured uniform examination of all participants. Additionally, PRISm and airflow definitions adhered to the GOLD 2025 guideline and data on spirometry and inflammatory markers was complete. Finally, the study was nationwide securing a large and representative sample of liver transplant recipients in Denmark.

In conclusion, in this large, well-described cohort of liver transplant recipients, elevated hs-CRP was associated with higher risk of PRISm and profoundly lower FEV_1_ and FVC. Longitudinal studies of the association between inflammatory markers and lung function decline in liver transplant recipients are required to provide further valuable insight into the role of systemic inflammation in the pathophysiology of lung function impairment in this immunosuppressed group.

## Data Availability

The data included in the current study are not publicly available as it contains information that could compromise participant privacy and consent. The data is available from the corresponding author upon reasonable request and with permission of the transplantation centers. Requests to access the datasets should be directed to susanne.dam.poulsen@regionh.dk.
